# Mixed effects analysis of factors associated with barriers to accessing healthcare among women in sub-Saharan Africa: Insights from demographic and health surveys

**DOI:** 10.1371/journal.pone.0241409

**Published:** 2020-11-09

**Authors:** Abdul-Aziz Seidu

**Affiliations:** 1 Department of Population and Health, College of Humanities and Legal Studies, University of Cape Coast, Cape Coast, Ghana; 2 College of Public Health, Medical and Veterinary Sciences, James Cook University, Townsville, Queensland, Australia; Iwate Medical University, JAPAN

## Abstract

**Background:**

Access to healthcare is one of the key global concerns as treasured in the Sustainable Development Goals. This study, therefore, sought to assess the individual and contextual factors associated with barriers to accessing healthcare among women in sub-Saharan Africa (SSA).

**Materials and methods:**

Data for this study were obtained from the latest Demographic and Health Surveys (DHS) conducted between January 2010 and December 2018 across 24 countries in SSA. The sample comprised 307,611 women aged 15–49. Data were analysed with STATA version 14.2 using both descriptive and multilevel logistic regression modelling. Statistical significance was set at p<0.05.

**Results:**

It was found that 61.5% of women in SSA face barriers in accessing healthcare. The predominant barriers were getting money needed for treatment (50.1%) and distance to health facility (37.3%). Women aged 35–39 (AOR = 0.945, CI: 0.911–0.980), married women (AOR = 0.694, CI: 0.658–0.732), richest women (AOR = 0.457, CI:0.443–0.472), and those who read newspaper or magazine at least once a week (AOR = 0.893, CI:0.811–0.983) had lower odds of facing barriers in accessing healthcare. However, those with no formal education (AOR = 1.803, CI:1.718–1.891), those in manual occupations (AOR = 1.551, CI: 1.424–1.689), those with parity 4 or more (AOR = 1.211, CI: 1.169–1.255), those who were not covered by health insurance (AOR = 1.284, CI: 1.248–1.322), and those in rural areas (AOR = 1.235, CI:1.209–1.26) had higher odds of facing barriers to healthcare access.

**Conclusion:**

Both individual and contextual factors are associated with barriers to healthcare accessibility in SSA. Particularly, age, marital status, employment, parity, health insurance coverage, exposure to mass media, wealth status and place of residence are associated with barriers to healthcare accessibility. These factors ought to be considered at the various countries in SSA to strengthen existing strategies and develop new interventions to help mitigate the barriers. Some of the SSA African countries can adopt successful programs in other parts of SSA to suit their context such as the National Health Insurance Scheme (NHIS) and the Community-based Health Planning and Services concepts in Ghana.

## Background

The health of women has been held in high esteem globally. This was prioritised by the erstwhile Millennium Development Goals (MDGs) and has been highlighted in its successor, the Sustainable Development Goals (SDGs) [1]. Specifically, SDG-3 targets 3.8 and 3.7 emphasize universal health coverage and access to sexual and reproductive healthcare services, including family planning information and education, and the integration of reproductive healthcare into national strategies and programmes by 2030 [2, 3]. SDG target 3.1 also aims at reducing maternal mortality to less than 70 per 100,000 live births by 2030 [4].

Despite the fact that these global targets have yielded positive results in terms of women’s health outcomes, massive improvements are still needed. In 2016, 303,000 women died from maternal-related causes [3, 5] and sub-Saharan Africa (SSA) recorded over 60% of these deaths [6]. Furthermore, in almost all countries globally, non-communicable diseases have also been among the major causes of death and disability among women, with higher rates for low- and middle-income countries [7]. Evidence again suggests that most women face barriers in their quest to accessing healthcare, which has resulted in poorer health outcomes such as miscarriage, unsafe abortions, and stillbirths [8].

Country-level studies such as those from Ethiopia [9, 10], Rwanda [11], Cameroon, and India [12] have revealed that individual and contextual factors are likely to obstruct women’s access to healthcare. Specifically, these barriers include transportation, geographical location, system organisational barriers, general availability of services, health information, waiting times and health infrastructure [13]. To achieve SDG 3, it is important to enhance universal access to health services that guarantee the health needs and aspirations of women of reproductive age. It is, therefore, prudent to get empirical evidence to provide a holistic understanding of the barriers to healthcare among women in SSA. This study, therefore, seeks to assess the individual and contextual factors associated with barriers in accessing healthcare among women in SSA.

## Materials and methods

### Data source

Data for this study were obtained from current Demographic and Health Surveys (DHS) conducted between January 1, 2010 and December 31, 2018 in 24 SSA countries (see Table 1). The choice of the 24 countries was influenced by the availability of the variables of interest in their datasets. DHS is a nationwide survey undertaken across low- and middle-income countries every five-year period[14]. The survey is representative of each of these countries and targets core maternal and child health indicators such as healthcare accessibility, unintended pregnancy, contraceptive use, skilled birth attendance, immunisation among under-fives, intimate partner violence, access to healthcare, and issues regarding men’s health such as tobacco and contraceptive use. In selecting the sample for each survey, multi-stage sampling approach was employed. The first step of this sampling approach involved the selection of clusters (i.e., enumeration areas [EAs]), followed by systematic household sampling within the selected EAs. In this study, the sample size consisted of women aged 15–49 who had complete information on all the variables of interest (N = 307,611). The Strengthening Reporting of Observational studies in Epidemiology (STROBE) guideline was used in the preparation of this manuscript [15]. The dataset is freely available for download at https://dhsprogram.com/data/available-datasets.cfm

**Table 1:** Sample size.

Country	Survey Year	Weighted Sample	Weighted Percentage
1. Benin	2017–2018	15,410	5.0
2. Burundi	2016–2017	16,783	5.5
3. Dr Congo	2013–2014	18,667	6.1
4. Ethiopia	2016	15,299	5.0
5. Gabon	2012	8,213	2.7
6. Ghana	2014	9,365	3.0
7. Gambia	2013	10,051	3.3
8. Guinea	2018	10,553	3.4
9. Kenya	2014	14,501	4.7
10. Liberia	2013	9,013	2.9
11. Lesotho	2014	2,849	0.9
12. Mali	2018	10,410	3.4
13. Malawi	2015–2016	24,540	8.0
14. Nigeria	2018	28,582	9.3
15. Niger	2012	11,023	3.6
16. Namibia	2013	9,100	3.0
17. Sierra Leone	2013	16,350	5.3
18. Chad	2014–2015	5,940	1.9
19. Togo	2013–2014	9,381	3.1
20. Tanzania	2015–2016	13,253	4.3
21. Uganda	2016	18,458	6.0
22. South Africa	2016	4,049	1.3
23. Zambia	2018	16,014	5.2
24. Zimbabwe	2015	9,809	3.2
Total	-	**307,611**	100.0

### Definition of variables

#### Outcome variable

The outcome variable in this study was barriers to healthcare accessibility. It was derived from four questions on barriers to healthcare access that each woman responded to. These focused on difficulty in obtaining money (money), distance to health facility (distance), getting permission for treatment (permission), and not wanting to go alone (companionship). If a woman faced at least one or more of the problems (money, distance, companionship, and permission), she was considered to have barriers to healthcare access and coded as “1”. However, if she did not report difficulty in getting money, distance, companionship, and permission-related barriers, she was considered not to have barriers to healthcare access and coded as “0” [16–18].

#### Independent variables

Both individual and contextual level factors were considered in this study. These variables were chosen based on their statistically significant association with barriers to healthcare access in previous studies [16–18]. The individual level factors included age (15–19, 20–24, 25–29, 30–34, 35–39, 40–44, 45–49), marital status (never married, married, cohabiting, widowed, divorced), educational level (no education, primary, secondary, higher), employment (not working, managerial, clerical, sales, house/domestic, agricultural, services, manual), parity (0,1–3, 4 or more), health insurance subscription (yes, no), and exposure to mass media, specifically, radio, newspaper, and television (not at all, less than once a week, at least once a week, almost every day). The contextual variables were sex of household head (male, female), household wealth status (poorest, poorer, middle, richer, richest), and type of residence (urban, rural) (see Table 2).

**Table 2:** Socio-demographic characteristics and barriers to health care access among women in SSA.

Variables	Weighted		Barrier in Healthcare Access		P-values
	Sample				
	N = 307, 611				
	n	%	No (%)	Yes (%)	
Individual level factors					
** Age**					p<0.001
** **15–19	61,599	20.0	39.0	61.0	
** **20–24	55,777	18.1	39.9	60.1	
** **25–29	54,677	17.8	40.0	60.0	
** **30–34	45,511	14.8	39.1	61.0	
** **35–39	38,719	12.6	38.3	61.7	
** **40–44	28,223	9.2	36.4	63.6	
** **45–49	23,106	7.5	34.7	65.3	
**Marital status**					p<0.001
** **Never married	80,822	26.3	43.4	56.6	
** **Married	168,425	54.8	38.4	61.6	
Cohabiting	30,783	10.0	33.2	66.8	
Widowed	8,444	2.8	30.3	69.7	
Divorced	19,136	6.2	33.8	66.2	
**Education**					p<0.001
** **No education	92,888	30.2	29.7	70.3	
** **Primary	99,495	32.3	33.2	66.9	
** **Secondary	98,832	32.1	47.8	52.2	
** **Higher	16,396	5.3	68.7	31.3	
**Employment**					p<0.001
Not working	100,209	32.6	39.8	60.2	
Managerial	14,048	4.6	63.1	36.9	
Clerical	2,989	1.0	67.6	32.4	
Sales	56,511	18.4	44.6	55.4	
House/domestic	6,601	2.2	46.9	53.1	
Agricultural	78,344	25.5	25.6	74.4	
Services	22,872	7.4	43.0	57.0	
Manual	26,036	8.5	38.9	61.1	
**Parity**					p<0.001
** **None	78,716	25.6	43.1	56.9	
** **1–3 children	120,208	39.1	41.6	58.4	
** **4 or more children	108,687	35.3	32.3	67.7	
**Health insurance coverage**					p<0.001
** **No	281,465	91.5	37.2	62.8	
** **Yes	26,146	8.5	54.8	45.2	
** Frequency of listening to radio**					p<0.001
Not at all	119,195	38.8	30.5	69.5	
** **Less than once a week	65,306	21.2	39.3	60.7	
** **At least once a week	113,563	36.9	46.3	53.8	
Almost every day	9,548	3.1	46.9	53.1	
** Frequency of reading newspaper or magazine**					p<0.001
** **Not at all	238,357	77.5	34.8	65.3	
** **Less than once a week	37,626	12.2	49.9	50.1	
** **At least once a week	29,126	9.5	55.9	44.1	
Almost every day	2,503	0.8	46.5	53.6	
** Frequency of watching television**					p<0.001
** **Not at all	180,039	58.5	29.3	70.7	
** **Less than once a week	41,284	13.4	43.9	56.1	
** **At least once a week	72,048	23.4	58.0	42.0	
Almost every day	14,240	4.6	45.2	54.8	
**Contextual factors**					
** Sex of household head**					p<0.001
** **Male	221,333	72.0	38.5	61.5	
** **Female	86,278	28.1	39.2	60.8	
** Wealth status**					p<0.001
** **Poorest	53,412	17.4	22.9	77.1	
** **Poorer	56,717	18.4	28.2	71.8	
** **Middle	59,132	19.2	34.3	65.8	
** **Richer	64,330	20.9	43.1	56.9	
** **Richest	74,021	24.1	57.8	42.2	
** Type of place of residence**					p<0.001
** **Urban	116,585	37.9	51.7	48.3	
** **Rural	191,026	62.1	30.8	69.2	

### Statistical analyses

The data were analysed with STATA version 14.2 for MacOS. Three basic steps were followed to analyse the data. The first step was the use of descriptive statistics to describe the sample and also cross-tabulation of all the independent variables against barriers to healthcare access. The second step was a bivariate analysis to select potential variables for the regression analysis. Variables that were statistically significant at the bivariate analysis at p<0.05 were moved to the regression stage, which involved a two-level multilevel binary logistic regression analyses to assess the individual and contextual factors associated with barriers to healthcare access. Clusters were considered as random effect to account for the unexplained variability at the community level [19]. Four models were fitted (see Table 3). Firstly, model I was an empty model and had no predictors (random intercept). Afterwards, the model II contained only the individual-level variables, model III contained only contextual level variables, while model IV contained both individual level and contextual level variables. For all models, adjusted odds ratios (AOR) and their associated 95% confidence intervals (CIs) were presented. These models were fitted by a STATA command “melogit” for the identification of predictors with the outcome variable. For model comparison, the log-likelihood ratio (LLR) and Akaike information criteria (AIC) test were used. Using the variance inflation factor (VIF), the multicollinearity test showed that there was no evidence of collinearity among the independent variables (Mean VIF = 1.51, Maximum VIF = 2.09 and Minimum VIF = 1.09). Sample weight (v005/1,000,000) was applied in all the analysis to correct for over- and under-sampling while the SVY command was used to account for the complex survey design and generalizability of the findings.

**Table 3:** Multilevel logistic regression of individual and contextual factors associated with barriers to healthcare among women in SSA.

Variables	Model I	Model II AOR [95%CI]	Model III AOR [95%CI]	Model IV AOR [95%CI]
**Individual level factors**				
**Age**				
15–19		1.099***		0.986
		[1.053–1.147]		[0.944–1.030]
20–24		1.096***		1.014
		[1.054–1.139]		[0.975–1.055]
25–29		1.031		0.986
		[0.994–1.069]		[0.950–1.023]
30–34		0.972		0.953**
		[0.938–1.007]		[0.920–0.988]
35–39		0.951**		0.945**
		[0.917–0.986]		[0.911–0.980]
40–44		0.978		0.972
		[0.941–1.016]		[0.935–1.011]
45–49		Ref		Ref
**Marital status**				
Never married		0.836***		0.834***
		[0.790–0.885]		[0.787–0.883]
Married		0.690***		0.694***
		[0.656–0.726]		[0.658–0.732]
Cohabiting		0.958		0.928*
		[0.906–1.014]		[0.876–0.984]
Widowed		Ref		Ref
Divorced		0.969		0.966
		[0.913–1.028]		[0.910–1.026]
**Education**				
No education		2.265***		1.803***
		[2.161–2.374]		[1.718–1.891]
Primary		1.988***		1.676***
		[1.900–2.080]		[1.601–1.756]
Secondary		1.570***		1.438***
		[1.504–1.638]		[1.378–1.501]
Higher		Ref		Ref
**Employment**				
Not working		1.552***		1.449***
		[1.429–1.685]		[1.334–1.573]
Managerial		1.186***		1.156**
		[1.086–1.295]		[1.058–1.263]
Clerical		Ref		Ref
Sales		1.323***		1.280***
		[1.218–1.438]		[1.178–1.391]
House/domestic		1.403***		1.409***
		[1.273–1.547]		[1.278–1.553]
Agricultural		2.275***		1.909***
		[2.093–2.473]		[1.755–2.075]
Services		1.537***		1.420***
		[1.411–1.674]		[1.303–1.548]
Manual		1.588***		1.551***
		[1.458–1.729]		[1.424–1.689]
**Parity**				
None		Ref		Ref
1–3		1.075***		1.016
		[1.044–1.106]		[0.987–1.046]
4+		1.343***		1.211***
		[1.296–1.391]		[1.169–1.255]
**Health insurance coverage**				
No		1.251***		1.284***
		[1.216–1.287]		[1.248–1.322]
Yes		Ref		Ref
**Frequency of listening to radio**				
Not at all		1.433***		1.399***
		[1.365–1.506]		[1.331–1.470]
Less than once a week		1.278***		1.296***
		[1.215–1.344]		[1.231–1.364]
** **At least once a week		1.155***		1.174***
		[1.100–1.214]		[1.117–1.234]
Almost every day		Ref		Ref
**Frequency of reading newspaper or magazine**				
Not at all		1.034		1.03
		[0.942–1.135]		[0.937–1.132]
Less than once a week		0.893*		0.896*
		[0.813–0.982]		[0.814–0.986]
** **At least once a week		0.880**		0.893*
		[0.800–0.968]		[0.811–0.983]
Almost every day		Ref		Ref
**Frequency of watching television**				
Not at all		1.151***		0.907***
		[1.101–1.204]		[0.866–0.950]
Less than once a week		0.778***		0.706***
		[0.741–0.816]		[0.673–0.742]
** **At least once a week		0.577***		0.598***
		[0.551–0.604]		[0.571–0.626]
Almost every day		Ref		Ref
**Contextual factors**				
**Sex of household head**				
Male			0.990	1.000
			[0.973–1.007]	[0.981–1.020]
Female			Ref	Ref
**Wealth status**				
Poorest			Ref	Ref
Poorer			0.730***	0.785***
			[0.710–0.749]	[0.764–0.806]
Middle			0.567***	0.658***
			[0.552–0.582]	[0.641–0.676]
Richer			0.435***	0.570***
			[0.424–0.447]	[0.554–0.586]
Richest			0.274***	0.457***
			[0.266–0.282]	[0.443–0.472]
**Place of residence**				
Urban			Ref	Ref
Rural			1.499***	1.235***
			[1.458–1.262]	[1.209–1.262
*N*		307,611	307,611	307,611
**Parameters**				
Community-level variance (SE)	0.43(0.022)	0.29(0.017)	0.34(0.019)	0.27(0.175)
ICC (%)	11.7%	8.1%	9.6%	8.2%
Log-likelihood	-201775.6	-189152.7	191013.06	-186503.04
LR Test	5866.45 (p<0.001)	3772.84 (p<0.001)	4522.01 (p<0.001)	3839.23 (p<0.001)
AIC	403555.2	378367.5	382042.1	373086.1
BIC	403576.5	378697.2	382127.2	373511.5

Exponentiated coefficients; 95% confidence intervals in brackets.

* *p* < 0.05

** *p* < 0.01

*** *p* < 0.001.

SE = Standard Error; ICC = Intra-Class Correlation; LR Test = Likelihood ratio Test; AIC = Akaike’s Information Criterion; BIC = Schwarz’s Bayesian Information Criteria.

Model I is the null model, a baseline model without any determinant variable.

Model II = individual level variables.

Model III = Contextual level variables.

Model IV is the final model adjusted for individual and Contextual level variables.

### Ethical approval

Ethical clearance was obtained from the Ethics Committee of ORC Macro Inc. as well as Ethics Boards of partner organisations of the various countries, such as the Ministries of Health. The DHS follows the standards for ensuring the protection of respondents’ privacy. Inner City Fund International ensures that the survey complies with the U.S. Department of Health and Human Services regulations for the respect of human subjects. The survey also reports that both verbal and written informed consent were obtained from the respondents. However, this was a secondary analysis of data and, therefore, no further approval was required for this study. Further information about the DHS data usage and ethical standards are available at http://goo.gl/ny8T6X.

## Results

### Prevalence of barriers to healthcare access

**Fig 1:**
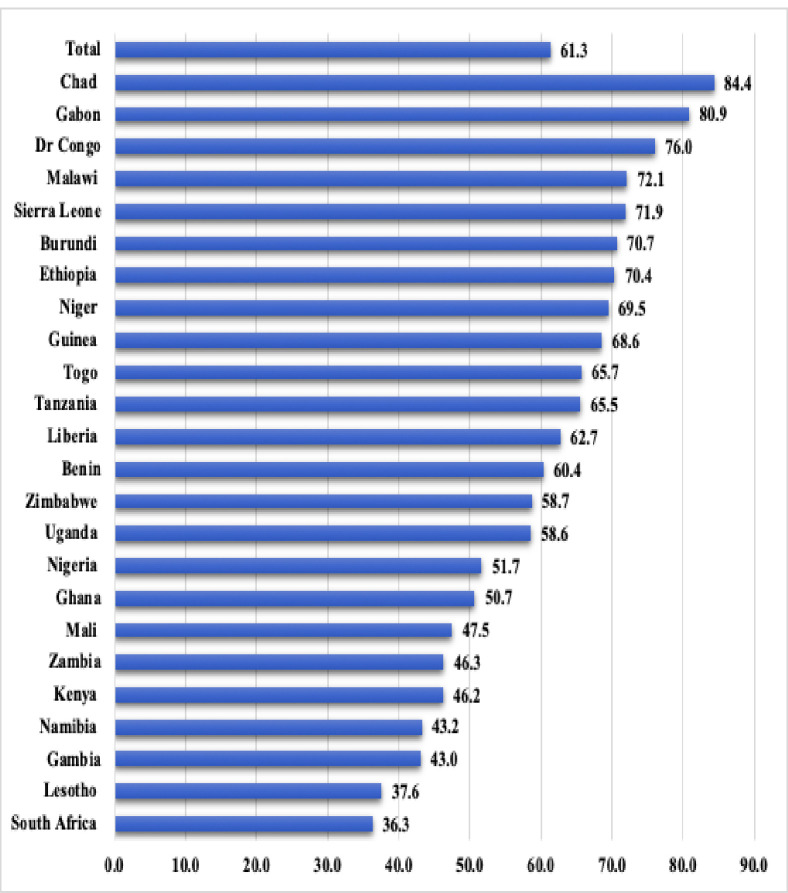
Prevalence of barriers to healthcare access among women in SSA (%).

**Fig 2:**
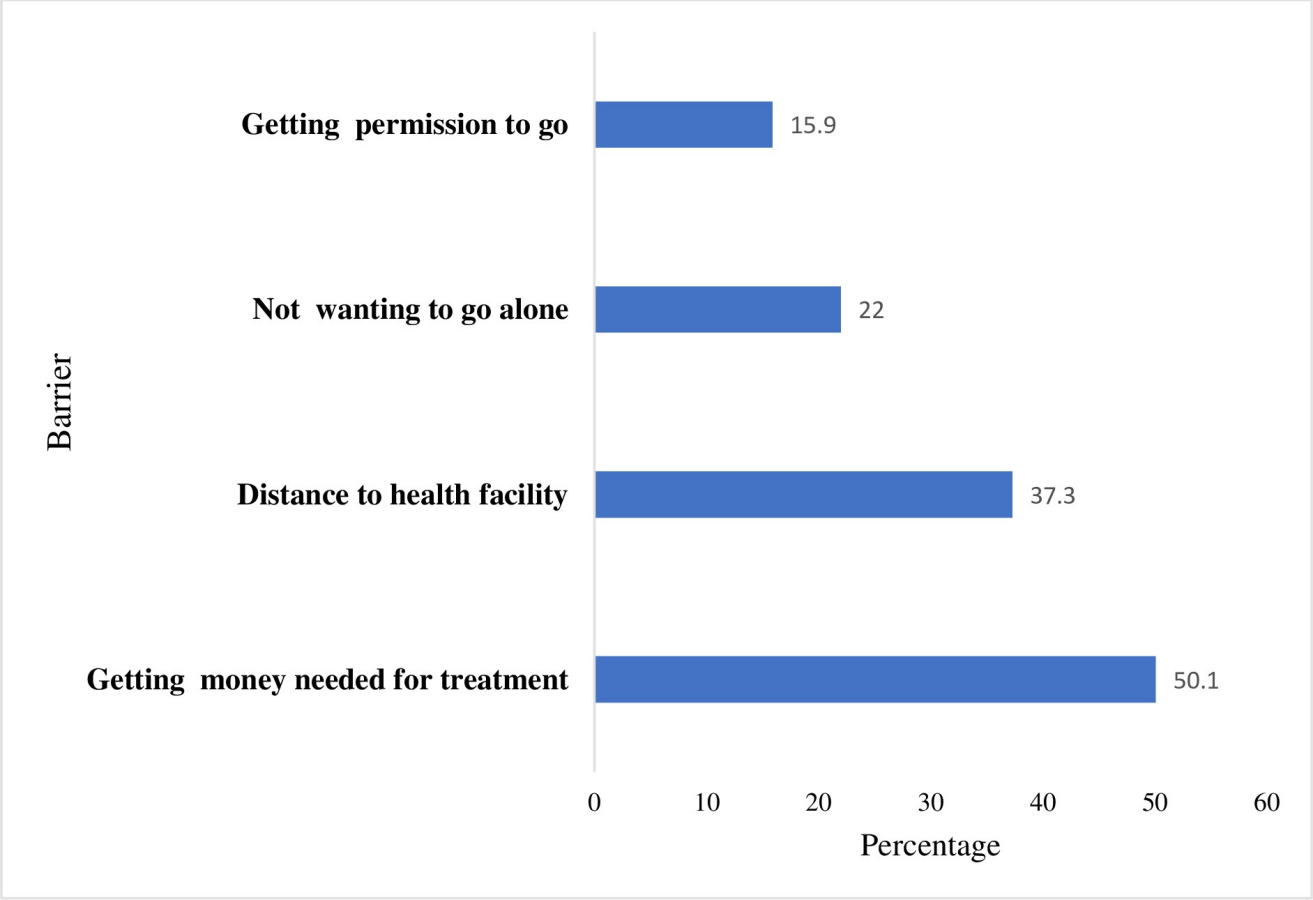
Types of barriers faced.

Figs 1 and 2 show the prevalence of barriers to healthcare access among women in SSA. From Fig 1, 61.5% of the women had at least one barrier in accessing healthcare. This ranged from 36.3% in South Africa to 84.4% in Chad. The major barrier these women faced was getting money needed for treatment (50.1%) and the least was getting permission to go (15.9%) (see Fig 2).

### Socio-demographic characteristics and barriers to healthcare access among women in SSA

Table 1 shows the socio-demographic characteristics and barriers to healthcare access among women in SSA. About 20% of the respondents were aged 15–19. More than half (54.8%) were married, 32.3% had primary level of education, 32.6% were not working, and 39.1% had 1–3 children. The greater percentage of the women were not covered by health insurance (91.5%). With access to mass media, 38.8%, 77.5%, and 58.5% were not exposed to radio, newspaper, and television respectively. The majority (72%) were in male-headed households, 24.1% were in the richest wealth quintile, and 62.1% were in rural areas. The Chi-square analysis showed that all the independent variables are associated with barriers to healthcare accessibility at p<0.05.

### Factors associated with barriers to healthcare access among women in SSA

Table 3 presents results on the factors associated with barriers in healthcare access among women in SSA. In terms of age, the result showed that women age 35–39 had the lowest odds in barriers to healthcare accessibility (AOR = 0.945, CI: 0.911–0.980), compared to those aged 45–49. In terms of marital status, married women had lower odds of facing barriers in healthcare accessibility (AOR = 0.694, CI: 0.658–0.732), compared to women who were widowed. Compared with women with higher level of education, those with no formal education had highest odds of facing barriers to healthcare accessibility (AOR = 1.803, CI:1.718–1.891). Regarding employment status, compared to those engaged in clerical works, those who are not working (AOR = 1.449, CI: 1.334–1.573), those engaged in agriculture (AOR = 1.909, CI = 1.755–2.075), and manual workers (AOR = 1.551, CI: 1.424–1.689) had higher odds of facing barriers to healthcare. In relation to parity, those with parity 4 or more [AOR = 1.211, CI: 1.169–1.255] had higher odds of facing barriers to healthcare. The result also showed that those who were not covered by health insurance had higher odds (AOR = 1.284, CI: 1.248–1.322) of barriers to healthcare accessibility, compared to those who were covered by health insurance. The result also showed that those who watched television at least once a week (AOR = 0.598, CI:0.571–0.626) and those read newspaper or magazine at least once a week(AOR = 0.893, CI:0.811–0.983) had lower odds of healthcare accessibility barriers, compared to those who watched television and read newspaper or magazine almost every day. With the contextual factors that were considered in the study, the result also showed that women in the richest wealth quantile had lower odds of facing barriers to healthcare, compared to women in the poorer wealth quantile (AOR = 0.457, CI:0.443–0.472). Those in rural areas (AOR = 1.235, CI:1.209–1.262) had higher odds of facing barriers to healthcare, compared with those in urban areas.

## Discussion

### Summary of main findings

This study sought to assess the individual and contextual factors associated with barriers to healthcare among women in SSA. The results showed that barriers to accessing healthcare is prevalent among women in SSA, with every 6 out of 10 women facing barriers in accessing healthcare. Getting money needed for healthcare and distance to healthcare are the major barriers. The individual factors associated with barriers to accessing healthcare are age, marital status, level of education, employment, parity, health insurance subscription, frequency of listening to radio, frequency of reading newspaper or magazine, and frequency of watching television. The contextual factors associated with barriers to healthcare are wealth status and place of residence.

### Synthesis with previous evidence

The prevalence in this study is similar to prevalence of healthcare accessibility barriers reported in South Africa (65%) [20], Rwanda (64%) [18], Ethiopia (69%) [17], and Tanzania (65%) [16]. The predominant barriers were getting money needed for treatment and distance to health facility. This confirms a previous study in Ethiopia by Tessema and Kebede [17]. The study also showed that women aged 30–34 and 35–39 had lower odds of facing barriers in accessing healthcare, compared to those aged 45 and above. This finding corroborates what has been observed in previous studies in other countries such as Nigeria [21] and Malaysia [22].

The study found that married women and those who had never married married had lower odds of facing a barrier to healthcare access, compared to the widowed. This confirms the findings of several empirical studies in other countries such as Southern Ethiopia [23], Tanzania[16], Afar Region of Ethiopia [24], Montenegro [25], and Malaysia [26].The probable explanation is that married women may gain economic and psychosocial support from their spouses to access healthcare [27].With the widowed women, in some parts of SSA, certain socio-cultural practices and customs deny them of befitting inheritance, social protection, and access to healthcare. Azah [28], for instance, opined that some widowhood rites in Ghana usually lead to women’s inability to inherit their partners’ property, which leaves them in abject poverty, marginalized, and unable to afford healthcare. Govender and Penn-Kekana [29] similarly alluded to the fact that unfavourable socio-cultural practices towards widows in many low- and middle-income countries inhibit them from accessing healthcare.

The study also showed that women with no formal education and those with low level of education had higher odds of experiencing barriers to healthcare. Relatedly, the study established that wealth as a contextual factor was also a significant determinant of barrier to healthcare access. Specifically, those in the richest wealth quintile had lowest odds of barrier to healthcare access. In addition, unemployed women had higher odds of facing barriers to healthcare access. Similar findings were reported in previous studies in Ghana [30], Tanzania [16], Uganda [31], Afghanistan [32], Ethiopia [10, 24], and Southern Mozambique [33]. Wealth, education, and employment are proxy measures of socio-economic status, which has been found to be associated with access to healthcare. Specifically, those in high socio-economic status may be in a better position to afford the cost associated with accessing healthcare, which is a common challenge among poorer women [34].The highly educated women are also likely to be in higher paid jobs and, as such, could easily afford healthcare no matter the cost and distance. The highly educated women, all things being equal, are also more informed regarding their fundamental human rights and may have higher health literacy. As a result, they are more likely to overcome any form of barrier to healthcare, compared to their counterparts who are less educated and may have lower health literacy, which has been found to be a key barrier to healthcare utilization [34]. High education and good job may give women the financial power and independence to enable them to afford healthcare, thereby overcoming the barrier of cost, distance, and decision-making [35].

Another key finding in this study is that women who were not covered by health insurance were more likely to face barriers in accessing healthcare. Theoretically, this finding could be argued within the context of the healthcare utilization model by Anderson and Newman [36], which stipulates that health insurance subscription is an enabling factor to healthcare accessibility. The finding also supports findings from previous studies in Ghana [37–39] which showed that health insurance ownership facilitates access to various maternal healthcare services.

It was also found that women who reside in rural areas had higher odds of barriers of healthcare access, compared to urban dwellers. This is in line with other studies in Ghana [30], Tanzania [16], and South Africa [20] which also found a higher likelihood of barriers to healthcare access in rural areas. The basic explanation could be that, in most parts of SSA, rural areas are less privileged manifesting in less health infrastructure, bad road network, and influence of socio-cultural practices that demand women to seek permission from their partners before seeking healthcare [17].

Exposure to mass media also showed decreased odds of healthcare accessibility barriers, which corroborates earlier studies in Ethiopia [40], India [41], Bangladesh [42], and rural Malawi [43]. The reason for this could be that listening to radio, reading newspaper, and watching television increase ones’ health literacy, which has been identified as a key enabler to healthcare utilization [44].

### Strengths and limitations of the study

The key strength of this study is the use of nationally representative data to assess individual and contextual factors associated with barriers to accessing healthcare among women in SSA. The findings can, therefore, be generalized to all women in their reproductive age in SSA. The study also employed advanced statistical models, which accounted for the clusters within the sample. Despite these strengths enumerated, the study design was cross-sectional and, therefore, causal interpretation cannot be deduced. Finally, due to the fact that secondary data was used, health-worker related factors could not be accounted for in this study.

## Conclusion

It was found that 61.5% of women face barriers in accessing healthcare in SSA. The major barriers were getting money needed for treatment and distance to health facility. Both individual and contextual factors were associated with barriers to healthcare accessibility. Particularly, age, marital status, employment, parity, health insurance coverage, frequency of listening to radio, frequency of reading newspaper or magazine, frequency of watching television, wealth status, and place of residence were associated with barriers to healthcare accessibility. These factors ought to be considered at the various countries in SSA to strengthen existing strategies and develop new interventions to help mitigate barriers to accessing healthcare among women. Specifically, some of the SSA African countries can adapt successful programs in other SSA countries to suit their context such as the National Health Insurance Scheme (NHIS) and the Community Health-based Planning Services (CHPs) concept in Ghana. There is also the need to empower women economically. These will aid in the achievement of the SDGs, 3.1, 3.7, and 3.8.

## Abbreviations

AOR: Adjusted Odds Ratio
CI: Confidence Interval
DHS: Demographic and Health Survey
VIF: Variance Inflation Factor
SDG: Sustainable Development Goals
LMICs: Low- and Middle-Income Countries
WHO: World Health Organisation
GSS: Ghana Statistical Service
LLR: log-likelihood ratio
SE: Standard Error
ICC: Intra-Class Correlation
LR Test: Likelihood ratio Test
AIC: Akaike’s Information Criterion
BIC: Schwarz’s Bayesian Information Criteria

## References

[1] United Nations 2015, *Transforming our World: The 2030 Agenda for Sustainable Development*, Geneva.

[2] Badiuzzaman M, Murshed SM, Rieger M. Improving maternal health care in a post conflict setting: evidence from Chittagong Hill tracts of Bangladesh. The Journal of Development Studies. 2020 Feb 1;56(2):384–400.

[3] World Health Organization. Primary health care on the road to universal coverage: 2019 global monitoring report WHO 2019

[4] Alkema L., Chou D., Hogan D., Zhang S., Moller A.B., Gemmill A., et al, 2016 Global, regional, and national levels and trends in maternal mortality between 1990 and 2015, with scenario-based projections to 2030: a systematic analysis by the UN Maternal Mortality Estimation Inter-Agency Group. *The Lancet*, 387(10017), pp.462–474. 10.1016/S0140-6736(15)00838-7 PMC5515236 26584737

[5] Regan L. Addressing unmet needs in global women’s health British Medical Association 2018

[6] WHO, World Health Statistics 2019: monitoring health for the SDGs sustainable development goals. 2019: World Health Organization. 2014

[7] WHO. Addressing the challenge of women’s health in Africa: Report of the commission on women’s health in Africa WHO 2014

[8] Mekonen AM, Gebregziabher MG, Teferra AS. The effect of community based health insurance on catastrophic health expenditure in Northeast Ethiopia: A cross sectional study. PloS one. 2018 Oct 18;13(10):e0205972 10.1371/journal.pone.0205972 30335838 PMC6193712

[9] Norton ME, Kuppermann M. Women should decide which conditions matter. 10.1016/j.ajog.2016.06.045 27793311

[10] Okwaraji YB, Webb EL, Edmond KM. Barriers in physical access to maternal health services in rural Ethiopia. BMC health services research. 2015 Jun;15(1):493 10.1186/s12913-015-1161-0 26537884 PMC4634737

[11] Nisingizwe MP, Tuyisenge G, Hategeka C, Karim ME. Are perceived barriers to accessing health care associated with inadequate antenatal care visits among women of reproductive age in Rwanda?. BMC pregnancy and childbirth. 2020 Dec 1;20(1):88 10.1186/s12884-020-2775-8 32041559 PMC7011379

[12] Piyasena MM, Murthy GV, Yip JL, Gilbert C, Zuurmond M, Peto T, et al Systematic review on barriers and enablers for access to diabetic retinopathy screening services in different income settings. PloS one. 2019;14(4). 10.1371/journal.pone.0198979 31013274 PMC6478270

[13] George S., Daniels K., & Fioratou E. (2018). A qualitative study into the perceived barriers of accessing healthcare among a vulnerable population involved with a community centre in Romania. *International journal for equity in health*, 17(1), 41 10.1186/s12939-018-0753-9 29615036 PMC5883264

[14] United Nations, Department of Economic and Social Affairs, Population Division. World mortality 2019: Highlights (ST/ESA/SER.A/432). 2019.

[15] Olayinka O. A., Achi O. T., Amos A. O., & Chiedu E. M. (2014). Awareness and barriers to utilization of maternal health care services among reproductive women in Amassoma community, Bayelsa State. *International Journal of nursing and midwifery*, 6(1), 10–

[16] Nisingizwe MP, Tuyisenge G, Hategeka C, Karim ME. Are perceived barriers to accessing health care associated with inadequate antenatal care visits among women of reproductive age in Rwanda?. BMC pregnancy and childbirth. 2020 Dec 1;20(1):88 10.1186/s12884-020-2775-8 32041559 PMC7011379

[17] Tessema ZT, Kebede FB. Factors associated with perceived barriers of health care access among reproductive-age women in Ethiopia: a Secondary data analysis of 2016 Ethiopian Demographic and Health Survey. 10.1186/s12913-020-05485-y 32711517 PMC7382096

[18] Nisingizwe MP, Tuyisenge G, Hategeka C, Karim ME. Are perceived barriers to accessing health care associated with inadequate antenatal care visits among women of reproductive age in Rwanda?. BMC pregnancy and childbirth. 2020 Dec 1;20(1):88 10.1186/s12884-020-2775-8 32041559 PMC7011379

[19] Solanke BL, Oyinlola FF, Oyeleye OJ, Ilesanmi BB. Maternal and community factors associated with unmet contraceptive need among childbearing women in Northern Nigeria. Contraception and reproductive medicine. 2019 Dec 1;4(1):11 10.1186/s40834-019-0093-1 31497311 PMC6717978

[20] Silal S.P., et al, Exploring inequalities in access to and use of maternal health services in South Africa. BMC health services research, 2012 12(1): p. 120 22613037 10.1186/1472-6963-12-120 PMC3467180

[21] Azuh DE, Fayomi OO, Yartey Ajayi L. Socio-cultural factors of gender roles in women’s healthcare utilization in Southwest Nigeria. Open Journal of Social Sciences, 2015;3:105–17.

[22] Lau A., Spark S., Smith T. M., Fairley K. C., Guy J. R., Donovan B., et al (2016). Socio-demographic and structural barriers to being tested for Chlamydia in general practice. The Medical Journal of Australia, 204 (3), 112 10.5694/mja15.00933 26866549

[23] Kea AZ, Tulloch O, Datiko DG, Theobald S, Kok MC. Exploring barriers to the use of formal maternal health services and priority areas for action in Sidama zone, southern Ethiopia. BMC pregnancy and childbirth. 2018 Dec 1;18(1):96 10.1186/s12884-018-1721-5 29649972 PMC5897996

[24] King R, Jackson R, Dietsch E, Hailemariam A. Barriers and facilitators to accessing skilled birth attendants in Afar region, Ethiopia. Midwifery. 2015 May 1;31(5):540–6. 10.1016/j.midw.2015.02.004 25745841

[25] Bojovic O, Medenica M, Zivkovic D, Rakocevic B, Trajkovic G, Kisic-Tepavcevic D, et al Factors associated with patient and health system delays in diagnosis and treatment of tuberculosis in Montenegro, 2015–2016. PloS one. 2018 Mar 9;13(3):e0193997 10.1371/journal.pone.0193997 29522545 PMC5844538

[26] Abd Wahab SN, Satar NM, Tumin M. Socio-demographic factors and structural barrier in accessing public clinics among the urban poor in Malaysia. Journal of Social Sciences and Humanities,2020,17(3),71–81

[27] Ani F, Abiodun O, Sotunsa J, Faturoti O, Imaralu J, Olaleye A. Demographic factors related to male involvement in reproductive health care services in Nigeria. The European Journal of Contraception & Reproductive Health Care. 2016 Jan 2;21(1):57–67. 25875130 10.3109/13625187.2015.1036856

[28] Azah FP. The Impact of Widowhood Rite on the Ghanaian Woman in the New Millennium:A Call to Action in the Christian Milieu. Bulletin 2017 Vol. 49, No. 2 January-February. Sedos49-1-2-017_47–2015 16/02/17 06:55 Pagina 2. https://sedosmission.org/article/the-impact-of-widowhood-rite-on-the-ghanaian-woman-in-the-new-millennium/. Retrieved on 1/05/2020 at 11:23GMT

[29] Govender V, Penn-Kekana L. Gender biases and discrimination: a review of health care interpersonal interactions. Global public health. 2008 Apr 1;3(S1):90–103. 10.1080/17441690801892208 19288345

[30] Badu E, Gyamfi N, Opoku MP, Mprah WK, Edusei AK. Enablers and barriers in accessing sexual and reproductive health services among visually impaired women in the Ashanti and Brong Ahafo regions of Ghana. Reproductive health matters. 2018 Nov 8;26(54):51–60. 10.1080/09688080.2018.1538849 30465631

[31] Kalule-Sabiti I, Amoateng AY, Ngake M. The effect of socio-demographic factors on the utilization of maternal health care services in Uganda. African Population Studies. 2014 Apr 29;28(1):515–25.

[32] Steinhardt LC, Waters H, Rao KD, Naeem AJ, Hansen P, Peters DH. The effect of wealth status on care seeking and health expenditures in Afghanistan. Health policy and planning. 2009 Jan 1;24(1):1–7. 10.1093/heapol/czn043 19060032

[33] Munguambe K, Boene H, Vidler M, Bique C, Sawchuck D, Firoz T, et al Barriers and facilitators to health care seeking behaviours in pregnancy in rural communities of southern Mozambique. Reproductive health. 2016 Jun 1;13(1):31 10.1186/s12978-016-0141-0 27356968 PMC4943506

[34] Berkman ND, Sheridan SL, Donahue KE, Halpern DJ, Crotty K. Low health literacy and health outcomes: an updated systematic review. Annals of internal medicine. 2011 Jul 19;155(2):97–107. 10.7326/0003-4819-155-2-201107190-00005 21768583

[35] Azah FP. The Impact of Widowhood Rite on the Ghanaian Woman in the New Millennium:A Call to Action in the Christian Milieu. Bulletin 2017 Vol. 49, No. 2 January-February. Sedos49-1-2-017_47–2015 16/02/17 06:55 Pagina 2. https://sedosmission.org/article/the-impact-of-widowhood-rite-on-the-ghanaian-woman-in-the-new-millennium/. Retrieved on 1/05/2020 at 11:23GMT

[36] Andersen R, Newman JF. Societal and individual determinants of medical care utilization in the United States. The Milbank Memorial Fund Quarterly. Health and Society. 1973 Jan 1:95–124. 4198894

[37] Blanchet NJ, Fink G, Osei-Akoto I. The effect of Ghana’s National Health Insurance Scheme on health care utilisation. Ghana medical journal. 2012;46(2):76–84. 22942455 PMC3426378

[38] Mensah J, Oppong JR, Schmidt CM. Ghana's National Health Insurance Scheme in the context of the health MDGs: An empirical evaluation using propensity score matching. Health Econ. 2010;19(S1):95–106. 10.1002/hec.1633 20730999

[39] Yaya S, Da F, Wang R, Tang S, Ghose B. Maternal healthcare insurance ownership and service utilisation in Ghana: analysis of Ghana demographic and health survey. Plos one. 2019 Apr 25;14(4):e0214841 10.1371/journal.pone.0214841 31022201 PMC6483336

[40] Woldeamanuel BT. Trends and Factors Associated with Healthcare Utilization for Childhood Diarrhea and Fever in Ethiopia: Further Analysis of the Demographic and Health Surveys from 2000 to 2016. Journal of Environmental and Public Health. 2020 Feb 18;2020. 10.1155/2020/8076259 PMC7049399 32148530

[41] Ghosh D. Effect of mothers' exposure to electronic mass media on knowledge and use of prenatal care services: a comparative analysis of Indian states. The Professional Geographer. 2006 Aug 1;58(3):278–93.

[42] Uddin M.F. Impact of Mass Media on Antenatal Care (ANC) Utilization in Bangladesh. Institute for Population and Social Research (IPSR), Mahidol University, 149 2009

[43] Zamawe CO, Banda M, Dube AN. The impact of a community driven mass media campaign on the utilisation of maternal health care services in rural Malawi. BMC pregnancy and childbirth. 2016 Dec 1;16(1):21 10.1186/s12884-016-0816-0 26819242 PMC4730729

[44] Collins JH, Bowie D, Shannon G. A descriptive analysis of health practices, barriers to healthcare and the unmet need for cervical cancer screening in the Lower Napo River region of the Peruvian Amazon. Women's Health. 2019 Dec;15:1745506519890969 10.1177/1745506519890969 31840562 PMC6918491

